# No beneficial effect of aerobic whole-body electromyostimulation on lower limbs strength and power – a randomized controlled trial

**DOI:** 10.1186/s13102-024-00931-4

**Published:** 2024-07-02

**Authors:** Anne Krause, Christoph Centner, Moritz Walther, Daniel Memmert, Nicolas Walser, Ramona Ritzmann

**Affiliations:** 1 Institute of Training and Computer Science, German Sport University Cologne, Cologne, Germany; 2 Biomechanics, Praxisklinik Rennbahn, Muttenz, Switzerland; 3 Department of Sport and Science, University of Freiburg, Freiburg, Germany

**Keywords:** wbEMS, Force-related capacities, Countermovement jump, Isokinetic muscle strength

## Abstract

**Background:**

Applying whole-body electromyostimulation (wbEMS) to voluntary activation of the muscle is known to impact motor unit recruitment. Thus, wbEMS as an additional training stimulus enhances force-related capacities. This study aimed to evaluate the mono- and multiarticular strength adaptations to a running intervention with wbEMS compared to running without wbEMS.

**Methods:**

In a randomized controlled trial (RCT), 59 healthy participants (32 female/ 27 male, 41 ± 7 years) with minor running experience conducted an eight-week running intervention (2x/ week à 20 min) with a wbEMS suit (EG) or without wbEMS (control group, CG). Maximal isokinetic knee extensor and flexor strength and jump height during countermovement jumps were recorded prior and after the intervention to assess maximal strength and power.

**Results:**

Following eight weeks of running, maximal isokinetic knee extension torque decreased significantly over time for both interventions (EG $\Delta$ -4%, CG $\Delta$ -4%; F(1, 44.14) = 5.96, *p* = 0.02, ${\upeta }$ = 0.12). No changes were observed for flexion torque (F(1, 43.20) = 3.93, *p* = 0.05, ${\upeta }$ = 0.08) or jump height (F(1, 43.04) = 0.32, *p* = 0.57, ${\upeta }$ = 0.01).

**Conclusions:**

The outcomes indicate that there is no additional effect over neuromuscular function adaptations with the inclusion of wbEMS during running training. Knee extensor strength is even slightly reduced which supports the principle of training specificity in regards to strength adaptation. We conclude that strength improvements cannot be achieved by running with wbEMS.

**Trial registration:**

German Clinical Trials Register, ID DRKS00026827, date 10/26/21.

**Supplementary Information:**

The online version contains supplementary material available at 10.1186/s13102-024-00931-4.

## Introduction

Whole-body electromyostimulation (wbEMS) is a training technology that stimulates multiple muscle groups simultaneously with regionally dedicated intensity [1]. It differs from conventional electromyostimulation (EMS), which is focused on a specific muscle group, while wbEMS is applied to the motor endplates of the skeletal muscles of the entire body. In its most common setting, wbEMS applies short stimulations of moderate to high intensity intermitted by short phases of rest to the muscle for about 20 min. wbEMS and EMS induced by artificial innervation of skeletal muscles have been used to either supplement or substitute voluntary muscle activation in sports and rehabilitation settings, for example, for re-education of muscle action, facilitation of muscle contraction, and maintenance or improvement of muscle mass and strength [2–8].

In previous years, wbEMS has been applied combined with a multitude of training regimens aiming to improve muscular strength performance [7–9]. After a period of 14–16 weeks, wbEMS plus resistance training revealed improvements of + 7–9% in leg extensor strength [10, 11] as well as in countermovement jump height [12]. However, there are just limited investigations focusing on the addition of superimposed wbEMS during high-intensity endurance-type exercises. It has been shown that EMS alters motor unit recruitment patterns in a nonselective manner [13]. Given that large motor units are strongly associated with high contraction velocities and force generation [14], but are rarely addressed during low-intensity aerobic exercise [15], it might be hypothesized that the superimposition of wbEMS increases the adaptive response of the neuromuscular system following long-term endurance training. In addition, conducting aerobic exercise such as running interferes with the development of muscular strength [16]. These generally small effects on neuromuscular adaptations following aerobic training have been argued to be related to the low level of (mechanical) loading during endurance-type training bouts, which are needed to facilitate alterations in neural drive to the musculature [17].

While evidence points towards positive effects of wbEMS in addition to resistance training in regard to strength-related parameters, to the knowledge of the authors, no investigation exists regarding the application of wbEMS during a running intervention. It is noticeable that most of the positive effects were mainly demonstrated in non-athletic study participants. Thus, the main objective of the present trial was to systematically investigate the effects of wbEMS during running on muscle strength performance in healthy individuals. Therefore, a longitudinal randomized-controlled trial was implemented comparing a running intervention with and without wbEMS on maximal isokinetic knee extension and flexion torque and countermovement jump performance. We hypothesized that following eight weeks of training, the application of wbEMS during running training facilitates significant improvement in mono- and multiarticular muscle strength and performance as compared to running training alone.

## Materials and methods

### Experimental design

In a parallel two-group randomized controlled design, long-term effects of an eight-week running intervention with and without wbEMS were investigated. Before and after the intervention, subjects were randomly assigned either to a group running without (control group, CG) or running with additional wbEMS (experimental group, EG). Participants were allocated with a random number generator. All outcome assessors were blinded to group allocation.

**Fig. 1:**
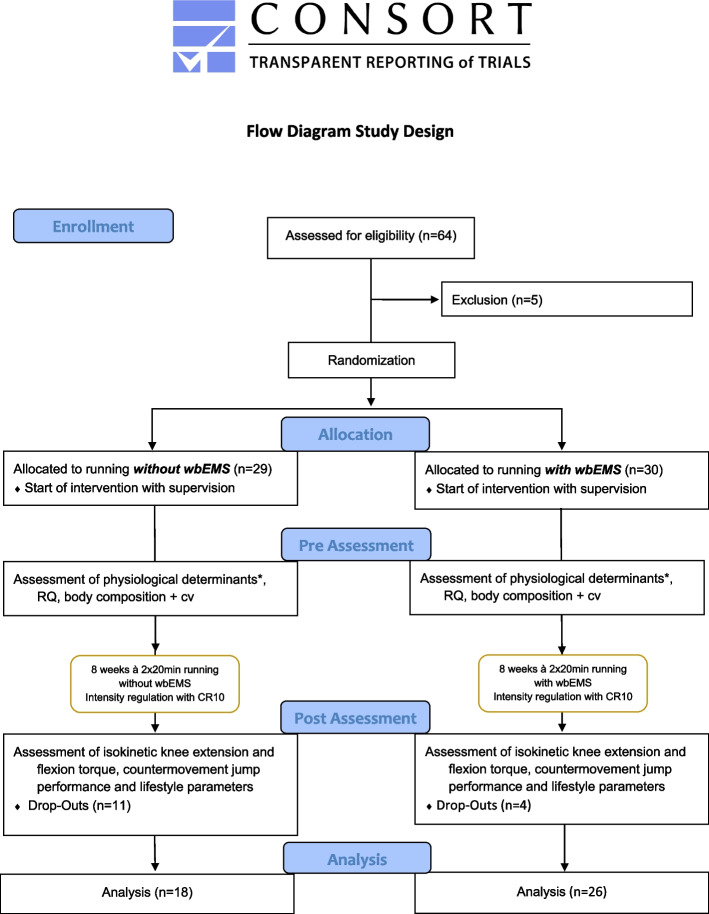
Change in maximum voluntary knee extension torque from pre (blue) to post (orange) for the intervention and control group. Dots indicate individual values

This study was conducted in accordance with the latest revision of the Declaration of Helsinki. All subjects gave written informed consent to the experimental procedure. This study was approved by the ethics committee of the German Sports University (001/2021). The study is registered in the German Clinical Trials Register with the ID DRKS00026827. Experimental procedures and potential risks were explained, and written informed consent was obtained before inclusion. The study adheres to CONSORT guidelines (Fig. 1).

### Participants

Recruitment period lasted from 08/2021 – 10/2021. Based on the results of a power analysis (G*Power V 3.1.9.2, power = 0.80, alpha = 0.05), 52 volunteers had to be recruited. Inclusion criteria comprised an age between 30 and 50, no or minor wbEMS (≤ once) and jogging-experience (< 4 km/ week). Participants during pregnancy, with neuronal, motor or metabolic diseases as well as with orthopedic injuries, cardiovascular or respiratory diseases, dermatological illnesses, physical disability limiting the participants’ mobility and medication which affects physical performance were excluded. Before commencing training, all subjects attended a preliminary screening, which included a medical anamnesis and physical examination to monitor agreement with the inclusion criteria. Participants were informed about contraindications [12, 18].

A total number of 59 healthy volunteers (32 female / 27 male, age 41 ± 7 years, body mass 73.7 ± 13.7 kg, height 173.6 ± 9.7 cm; rel. V’O_2max_ 40.2 ± 7.4 ml/min/kg) participated in the current study. Since we expected a drop-out rate of around 10% during the Covid pandemic, the number of subjects who started the study was 7 people higher than required by the power analysis. Fifteen participants (EG: 4, CG: 11) dropped-out during the intervention due to the following reasons: Covid-19 (5), injuries (5) and personal reasons (5). A flow chart as well as anthropometric details are described in Fig. 1 and in Krause et al. 2023 [19].

### Training

Both groups ran twice weekly for eight weeks either with (EG) or without wbEMS (CG). Each training was limited to 20 min [18] and there was at least 48 h of rest between two sessions [20]. Training intensity was increased based on the results of the initial incremental step test every two weeks and controlled by means of perceived exertion (rating of perceived exertion > 7 out of 10, Supplemental Table 1) as described elsewhere [19].

In Krause et al. (2023) the procedure for wbEMS (Xenoma, Japan) is described in detail. The stimulation parameters were defined according to current scientific guidelines in order to ensure the safety of the participants and to rule out muscle spasms, co-activation or disabilities during motion [8, 18, 21, 22]. Specifications were as follows: 26 bipolar electrodes transferred the electrical current to the muscles of the back (4), chest (4), abdomen (4), hip (2), thigh (8) and arms (4). Bipolar impulse type, stimulation sequencing 85 Hz, single stimulus width 180µs, rise 700 ms, duty cycle 50% on and 50% off (3 s on- and 3 s off-time). The appropriate current was determined individually for each participant and each session at a subjective tolerance rating of CR10-scale 7/10 [12].

Training sessions of all individuals were controlled and documented by the operators of the study. Individual training and suit stimulus intensity were adjusted and controlled for each participant by trained personnel during all times throughout the training process. All stimulation parameters were documented in the protocol.

### Assessments and outcome measures

Before and after the training period, assessments of isokinetic knee extensor- and flexor strength and explosive leg chain strength were conducted.

#### Isokinetic knee extension and flexion torque

An isokinetic dynamometer (Humac®/NormTM Testing & Rehabilitation System, Computer Sports Medicine, Inc. CSMi, Stoughton, Massachusetts, US) was used to assess knee extensor and flexor strength according to Li et al. [23]. The dynamometer was calibrated prior to testing sessions.

For the initial position, subjects were seated upright with the trunk at 100° and fixed by straps [22, 23]. The knee joint was in line with the mechanical axis of the dynamometer and the shin pad was placed superior to the medial malleolus as described elsewhere [23].

Prior to each test sequence subjects performed three submaximal repetitions with 50–60% estimated MVC to familiarize with the testing procedure. For data assessment, the protocol from Li and colleagues [23] was used which consisted of concentric-concentric contractions at 60°/s angular speed in the full individual range of motion (ROM). This has proven to be very reliable in terms of test–retest reliability [24]. Two sets of three repetitions with maximum effort were executed for the knee extensor and knee flexor muscles with a minimum of 3 min rest between repetitions. The trial with the highest torque values averaged for both legs were used for further analysis.

#### Countermovement jump

Subjects performed three countermovement jumps on a force plate (SP Sportdiagnosegeräte GmbH, Trins, Austria) to assess changes in lower limb power. Before the countermovement jumps, participants underwent a familiarization protocol. They were instructed to place their feet hip-width apart, to keep their knees in line with their feet, to rest both hands on the hips and to look straight ahead. The jump was initiated by the subjects lowering their center of mass to a limited decline depth between 25-35 cm. They were asked to jump as high as possible with their legs straightened during flight time. Out of three jumps, the maximal jump height was used for further analysis.

#### Lifestyle parameters

The participants were instructed to maintain their habitual dietary intake and normal physical activity during the study. To control for potential effects of activities performed outside the study, physical activity was assessed before and after the intervention using a validated questionnaire [25]. Subjects were also advised to record their dietary intake on 3 days (2 weekdays, 1 weekend day). The nutritional logs were analyzed with Nutriguide 4.6 [Nutri Science GmBH].

### Statistics

All values are presented as mean ± standard deviations and standard errors. Maximum force values are evaluated in relation to the respective body weight. The average differences between left and right leg were calculated and are described for each intervention group and time point.

For the inference analysis, a linear mixed model ANOVA (within-subject factor: time [2] x between-subject factor: group [2]) was calculated with the lmerTest package [26]. Level of significance was set at *p* < 0.05. Assumptions such as outliers, normality (Shapiro–Wilk test), homogeneity of variances (Levene test), assumption of sphericity (Mauchly’s test of sphericity) and homogeneity of covariances (Box’s m) were reviewed. Effect sizes and 95% confidence intervals were defined with generalized ${\eta }$ with reference values as follows: 0.01 = small, 0.06 = medium, 0.14 = large effect sizes.

Tukey correction for multiple testing was used for Post-Hoc tests. All statistical analyses were performed with the statistical software R version 1.4.1717.

## Results

### Maximum isokinetic knee extension and flexion torque

All descriptive and inference values of maximum isokinetic knee extension and flexion torque (T_max_) are described in Table 1. T_max_ decreased significantly in extensors over time for EG by -4 ± 13% and for CG by -4 ± 9% (F(1, 44.14) = 5.96, *p* = 0.02, ${\eta }$= 0.12). Post-Hoc tests revealed significant interaction post values (t(1,73.70) = 2.09, *p* = 0.04). For the flexors, no changes in T_max_ were observed (Table 1 and Fig. 1).

**Table 1:** Maximum torque values (T_max_) for the knee extension and knee flexion torque from pre- to post-intervention (mean ± SE)

Groups				**mixed ANOVA**		
		**EG**	**CG**	**Main time effect**	**Main group effect**	**Time and group interaction effect**
T_max extensors_ [NM % kg]	pre	213 ± 9	193 ± 2	F = 5.96 *p* = 0.02^*^ ${\eta }^{2}$=0.12	F = 4.05 *p* = 0.10 ${\eta }^{2}$=0.07	F = 0.56 *p* = 0.04^*^ ${\eta }^{2}$=0.01
	post	204 ± 9	190 ± 7			
T_max flexors_ [NM % kg]	pre	141 ± 7	131 ± 5	F = 3.93 *p* = 0.05 ${\eta }^{2}$=0.08	F = 1.61 *p* = 0.21 ${\eta }^{2}$=0.03	F = 0.00 *p* = 1.00 ${\eta }^{2}$=0.00
	post	144 ± 7	141 ± 5			

*EG* Experimental group, *CG* Control group, *T*_*max*_ Maximal flexion and extension torque, *p<0.05

### Counter movement jump

**Fig. 2:**
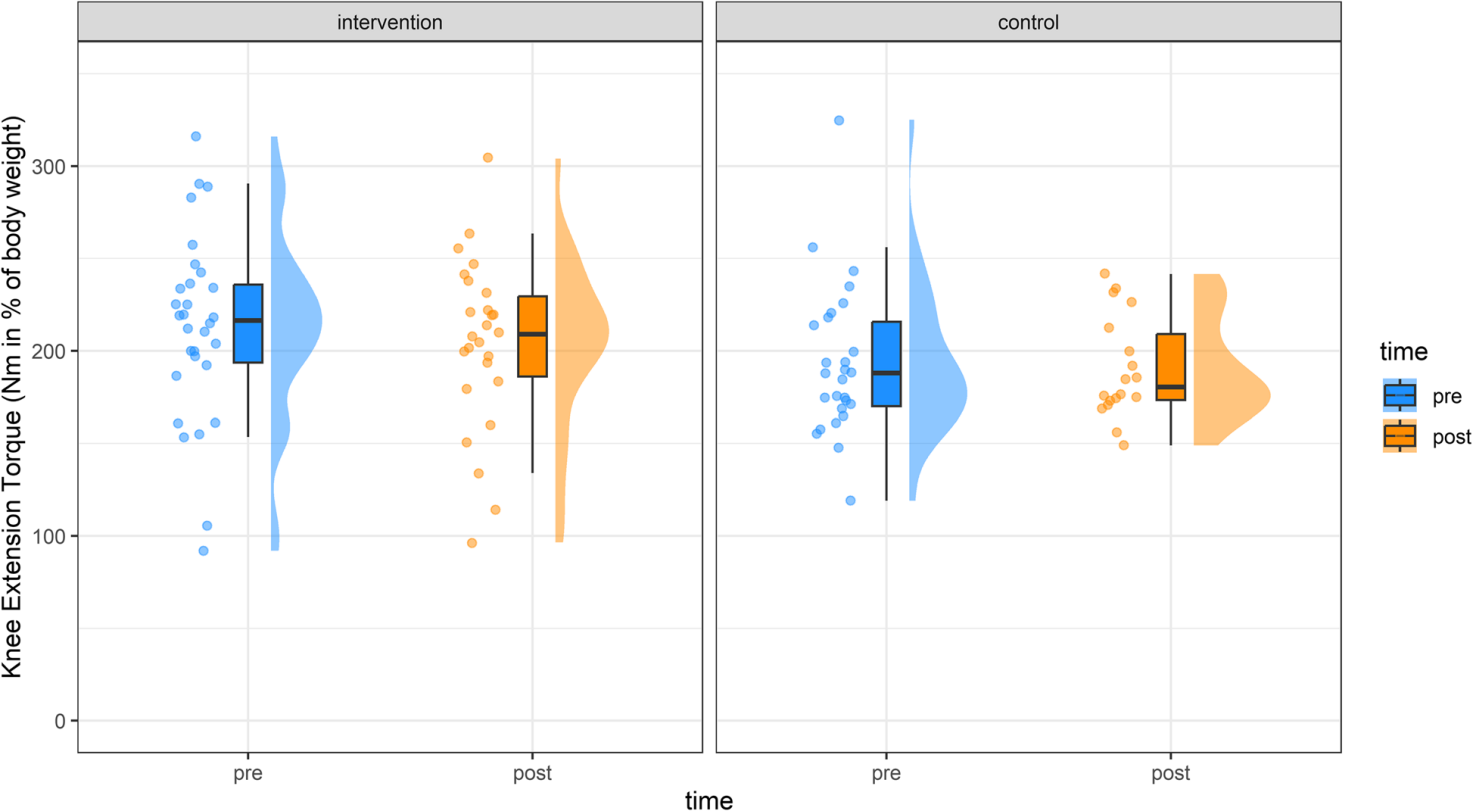
Knee extension torque from pre (blue) to post (orange) for the intervention and control group. Dots indicate individual values

**Fig. 3:**
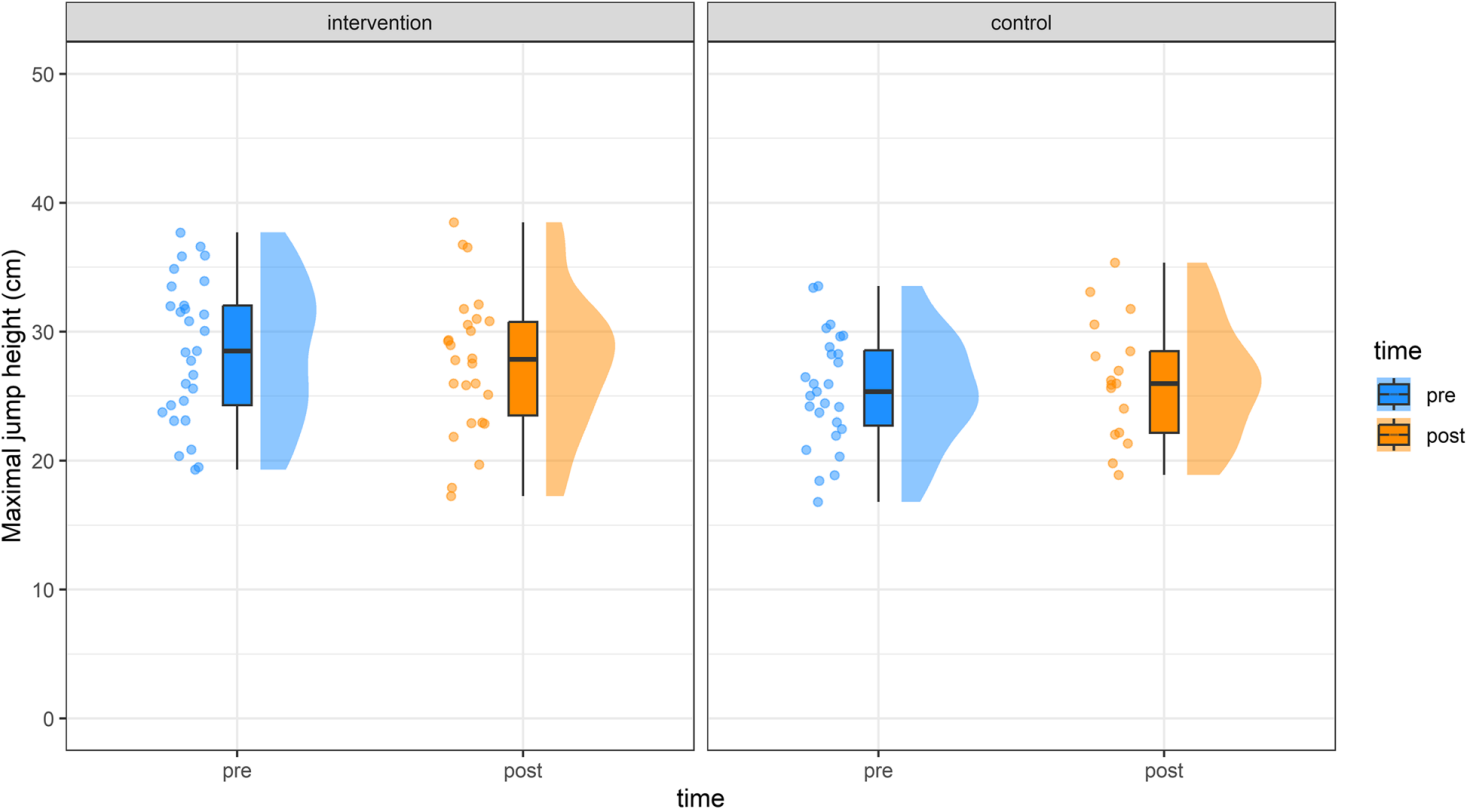
Maximum jump height from pre (blue) to post (orange) for the intervention and control group. Dots indicate individual values

Jump height differed significantly between groups prior to the intervention (F(1, 54.31) = 4.52, *p* = 0.04). However, no significant differences occurred over time or for interaction (Table 2 and Figs. 2 and 3).

**Table 2:** Jump height from pre- to post-intervention (mean ± SE)

Groups			**mixed ANOVA**		
	**EG**	**CG**	**Main time effect**	**Main group effect**	**Time and group interaction effect**
Height [cm] pre	28.6 ± 1.0	25.5 ± 0.8	F = 0.32 *p* = 0.57 ${\eta }^{2}$=0.01	F = 4.52 *p* = 0.04^*^ ${\eta }^{2}$=0.08	F = 0.42 *p* = 0.52 ${\eta }^{2}$=0.01
Height [cm] post	27.6 ± 1.1	26.3 ± 1.1			

*EG* Experimental group, *CG* Control group, *p<0.05

### Lifestyle parameters

Neither physical activity behavior throughout the week, nor caloric intake did change over time for both groups (Supplemental Table 2).

## Discussion

The findings of the present study revealed that an eight-week running training with wbEMS did not facilitate beneficial effects in muscular knee flexor strength or power in recreationally active individuals. Extensor strength was slightly reduced in both groups by 4% which is why the current hypothesis could not be verified.

*First,* the demonstrated effects occurred for both training groups. Therefore, it can be assumed that not the wbEMS stimulus itself but the type of training led to the fact that no strength-related improvements in performance were found. Interestingly, previous investigations comparing wbEMS to a high-intensity resistance training have shown that effects on strength parameters did not differ when wbEMS was compared to a high-intensity strength training [11]. In contrast to the current results, however, both groups gained leg extensor strength [11] instead of reducing it. A possible explanation might be training-specific stimuli [27]: wbEMS plus voluntary activation during resistance training was shown to enhance muscle strength [1, 6] or added during jump training to improve jump performance [4, 12]. Combining endurance and resistance training with wbEMS, such as in concurrent training, might be a valuable option to enhance endurance and strength performance [17, 28].

*Second,* while studies with local EMS have consistently demonstrated to improve muscle function [8], the evidence regarding wbEMS is conflicting with some studies reporting significant strength gains [1] of around 7% [11] to 9% [10] for leg extension and other demonstrating no changes [29]. One explanation could be found in the target group: minor but all the more relevant performance changes are more difficult to demonstrate in athletic participants [1, 29]. In addition to that, wbEMS can be used in a much more targeted manner during muscle contraction during resistance training. During running, a synchronized stimulation pattern is not applicable. Since various muscle groups (e.g., extensors and flexors of different leg segments from foot contact to toe off) are activated during running, the superimposed wbEMS might have led to increased agonist–antagonist co-activations and thus a poor functionality [29, 30], which is insufficient for the development of functional improvements. Thus, the movement itself is not sufficient to produce strength benefits either after resistance [1] or after running training with wbEMS.

Although not assessed in the current study, data from previous trials indicated that a general promotion of neuromuscular adaptability can in theory be achieved by EMS via activation of larger motor units at low force levels [13]. With an application of EMS over a muscle belly, a superficial-to-deep motor unit (MU) recruitment can be observed regardless of the MU size [31]. The extent to which larger MUs were activated with the wbEMS suits and running intensities in the present trial and whether this is associated with intra-individual strength improvements remains unanswered with the assessment methods used. However, if large MUs and superficial-to-deep MU recruitment is achieved with wbEMS during running, the current results clearly show that neurophysiological stimuli throughout wbEMS have no functional consequence in terms of multi- and monoarticular joint control.

*Third,* and with regard to the chosen intervention wbEMS during running, there are important aspects to consider: Previous findings indicated that wbEMS parameters (e.g., stimulation intensity, pulse frequency) are of high importance to induce optimal neuromuscular adaptations [12]. For triggering response to the muscular level, intensity-dependent adaptations can be observed with higher wbEMS intensities eliciting higher MU recruitment and evoke higher forces [32]. In the current trial, high frequencies (85 Hz) and short duty cycles (3 s “on”, 3 s “off”) were used. Although higher frequencies might be effective in activating both motor and sensory axons, and thus, maximizing the afferent signal [33], literature indicates that the application of high frequencies with wbEMS decreases motor axon excitability [34]. The extent to which this modulates long-term neuromuscular adaptation is unclear and requires further investigation.

## Conclusion

The addition of wbEMS to running training does not affect neuromuscular function parameters with reference to maximal lower extremity flexion and extension torque. Despite the eight-week intervention, wbEMS did not facilitate beneficial changes in muscular isokinetic knee flexion and extension torques or countermovement jump performance in recreationally active individuals. In the future, it might be valuable to investigate effects of wbEMS when muscles are activated selectively and in non-athletic participants. Based on these outcomes, we conclude that it is not sufficient to activate wbEMS during running training if the goal is to improve strength.

### Supplementary Information

Supplementary Material 1.

Supplementary Material 2.

## Data Availability

Data is available up on request from the corresponding author.

## References

[1] Kemmler W, Shojaa M, Steele J, Berger J, Fröhlich M, Schoene D (2021). Efficacy of Whole-Body Electromyostimulation (WB-EMS) on Body Composition and Muscle Strength in Non-athletic Adults. A Systematic Review and Meta-Analysis. Front Physiol..

[2] Amaro-Gahete FJ, De-la-O A, Sanchez-Delgado G, Robles-Gonzalez L, Jurado-Fasoli L, Ruiz JR (2018). Functional Exercise Training and Undulating Periodization Enhances the Effect of Whole-Body Electromyostimulation Training on Running Performance. Front Physiol..

[3] Amaro-Gahete FJ, De-la-O A, Sanchez-Delgado G, Robles-Gonzalez L, Jurado-Fasoli L, Ruiz JR (2018). Whole-Body Electromyostimulation Improves Performance-Related Parameters in Runners. Front Physiol..

[4] Filipovic A, Grau M, Kleinöder H, Zimmer P, Hollmann W, Bloch W (2016). Effects of a Whole-Body Electrostimulation Program on Strength, Sprinting, Jumping, and Kicking Capacity in Elite Soccer Players. J Sports Sci Med.

[5] Filipovic A, Kleinöder H, Plück D, Hollmann W, Bloch W, Grau M (2015). Influence of Whole-Body Electrostimulation on Human Red Blood Cell Deformability. J Strength Cond Res.

[6] Wirtz N, Zinner C, Doermann U, Kleinoeder H, Mester J (2016). Effects of Loaded Squat Exercise with and without Application of Superimposed EMS on Physical Performance. J Sports Sci Med.

[7] Currier DP, Mann R (1983). Muscular Strength Development by Electrical Stimulation in Healthy Individuals. Phys Ther.

[8] Filipovic A, Kleinöder H, Dörmann U, Mester J (2012). Electromyostimulation–a systematic review of the effects of different electromyostimulation methods on selected strength parameters in trained and elite athletes. J Strength Cond Res.

[9] Requena Sánchez B, Padial Puche P, González-Badillo JJ (2005). Percutaneous Electrical Stimulation in Strength Training: An Update. J Strength Cond Res.

[10] Kemmler W, Schliffka R, Mayhew JL, von Stengel S (2010). Effects of whole-body electromyostimulation on resting metabolic rate, body composition, and maximum strength in postmenopausal women: the Training and ElectroStimulation Trial. J Strength Cond Res.

[11] Kemmler W, Teschler M, Weißenfels A, Bebenek M, Fröhlich M, Kohl M (2016). Effects of Whole-Body Electromyostimulation versus High-Intensity Resistance Exercise on Body Composition and Strength: A Randomized Controlled Study. Evid Based Complement Alternat Med..

[12] Berger J, Ludwig O, Becker S, Backfisch M, Kemmler W, Fröhlich M (2020). Effects of an Impulse Frequency Dependent 10-Week Whole-body Electromyostimulation Training Program on Specific Sport Performance Parameters. J Sports Sci Med.

[13] Gregory CM, Bickel CS (2005). Recruitment patterns in human skeletal muscle during electrical stimulation. Phys Ther.

[14] Stifani N. Motor neurons and the generation of spinal motor neuron diversity. Front Cell Neurosci. 2014;8. Verfügbar unter: http://journal.frontiersin.org/article/10.3389/fncel.2014.00293/abstract. [zitiert 26. Juli 2023]. 10.3389/fncel.2014.00293 PMC4191298 25346659

[15] Vila-Chã C, Falla D, Farina D (2010). Motor unit behavior during submaximal contractions following six weeks of either endurance or strength training. J Appl Physiol.

[16] Hickson RC (1980). Interference of strength development by simultaneously training for strength and endurance. Eur J Appl Physiol.

[17] Hughes DC, Ellefsen S, Baar K (2018). Adaptations to Endurance and Strength Training. Cold Spring Harb Perspect Med.

[18] Kemmler W, Froehlich M, von Stengel S, Kleinöder H (2016). Whole-Body Electromyostimulation – The Need for Common Sense! Rationale and Guideline for a Safe and Effective Training. Dtsch Z Für Sportmed.

[19] Krause A, Walser N, Centner C, Memmert D, da Mota De Moreia I, Ritzmann R (2023). Running with whole-body electromyostimulation improves physiological determinants of endurance performance – a randomized control trial. BMC Sports Sci Med Rehabil..

[20] Stöggl T, Sperlich B (2014). Polarized training has greater impact on key endurance variables than threshold, high intensity, or high volume training. Front Physiol.

[21] Amaro-Gahete FJ, De-la-O A, Jurado-Fasoli L, Dote-Montero M, Gutiérrez Á, Ruiz JR (2019). Changes in Physical Fitness After 12 Weeks of Structured Concurrent Exercise Training, High Intensity Interval Training, or Whole-Body Electromyostimulation Training in Sedentary Middle-Aged Adults: A Randomized Controlled Trial. Front Physiol..

[22] Hainaut K, Duchateau J (1992). Neuromuscular electrical stimulation and voluntary exercise. Sports Med Auckl NZ.

[23] Li RC, Wu Y, Maffulli N, Chan KM, Chan JL (1996). Eccentric and concentric isokinetic knee flexion and extension: a reliability study using the Cybex 6000 dynamometer. Br J Sports Med.

[24] Sole G, Hamrén J, Milosavljevic S, Nicholson H, Sullivan SJ (2007). Test-Retest Reliability of Isokinetic Knee Extension and Flexion. Arch Phys Med Rehabil.

[25] Frey I, Berg A, Grathwohl D, Keul J (1999). Freiburger Fragebogen zur körperlichen Aktivität-Entwicklung. Prüfung und Anwendung Soz- Präventivmedizin SPM.

[26] Kuznetsova A, Brockhoff PB, Christensen RHB. lmerTest package: tests in linear mixed effects models. J Stat Softw. 2017;82(13). Verfügbar unter: http://www.jstatsoft.org/v82/i13/. [zitiert 28. Februar 2023].

[27] Häkkinen K, Mero A, Kauhanen H (1989). Specificity of endurance, sprint and strength training on physical performance capacity in young athletes. J Sports Med Phys Fitness.

[28] Gäbler M, Prieske O, Hortobágyi T, Granacher U (2018). The Effects of Concurrent Strength and Endurance Training on Physical Fitness and Athletic Performance in Youth: A Systematic Review and Meta-Analysis. Front Physiol.

[29] Wirtz N, Dörmann U, Micke F, Filipovic A, Kleinöder H, Donath L (2019). Effects of Whole-Body Electromyostimulation on Strength-, Sprint-, and Jump Performance in Moderately Trained Young Adults: A Mini-Meta-Analysis of Five Homogenous RCTs of Our Work Group. Front Physiol.

[30] Maffiuletti NA, Dirks ML, Stevens-Lapsley J, McNeil CJ (2023). Electrical stimulation for investigating and improving neuromuscular function in vivo: Historical perspective and major advances. J Biomech.

[31] Okuma Y, Bergquist AJ, Hong M, Chan KM, Collins DF (2013). Electrical stimulation site influences the spatial distribution of motor units recruited in tibialis anterior. Clin Neurophysiol.

[32] Blazevich AJ, Collins DF, Millet GY, Vaz MA, Maffiuletti NA (2021). Enhancing Adaptations to Neuromuscular Electrical Stimulation Training Interventions. Exerc Sport Sci Rev.

[33] Bergquist AJ, Clair JM, Collins DF (2011). Motor unit recruitment when neuromuscular electrical stimulation is applied over a nerve trunk compared with a muscle belly: triceps surae. J Appl Physiol.

[34] Luu MJ, Jones KE, Collins DF (2021). Decreased excitability of motor axons contributes substantially to contraction fatigability during neuromuscular electrical stimulation. Appl Physiol Nutr Metab.

