# Supplementary material for: No beneficial effect of aerobic whole-body electromyostimulation on lower limbs strength and power – a randomized controlled trial
Source: BMC Sports Sci Med Rehabil. 2024 Jul 2;16:144. doi: 10.1186/s13102-024-00931-4 (PMC11218063; doi:10.1186/s13102-024-00931-4)
Supplement: Supplementary file 1 — Supplementary Material 1. [file 13102_2024_931_MOESM1_ESM.docx]

Supplement Table 1: Intervention protocol

|  | Week 1&2 | | Week 3&4 | | Week 5&6 | | Week 7&8 | |
| --- | --- | --- | --- | --- | --- | --- | --- | --- |
|  | **Session 1** | **Session 2** | **Session 1** | **Session 2** | **Session 1** | **Session 2** | **Session 1** | **Session 2** |
| Duration [min] | 20’ | 20’ | 20’ | 20’ | 20’ | 20’ | 20’ | 20’ |
| Protocol | 20’ vLT1 | 2 x 4’ max  2’ rest | 15’ vLT1  5’ vLT2 | 2 x 4’ max  2’ rest | 10’ vLT1  10’ vLT2 | 3 x 4’ max  2’ rest | 3 x 4’ max  2’ rest | 3 x 4’ max  2’ rest |
| Frequency [Hz] | 85 | 85 | 85 | 85 | 85 | 85 | 85 | 85 |
| Duty cycle [%] | 50% (3:3) | 50% (3:3) | 50% (3:3) | 50% (3:3) | 50% (3:3) | 50% (3:3) | 50% (3:3) | 50% (3:3) |
| Median CR10 stimulus intensity | 6.3 | 7.0 | 7.0 | 7.0 | 7.0 | 7.0 | 7.0 | 7.0 |
| Median CR10 training intensity (EG / CG) | 3 (4 / 2) | 8 (8 / 8) | 6 (6 / 4) | 8 (8 / 8.5) | 6.5 (7 / 6) | 8 (8/ 8.3) | 8 (8 / 8) | 8 (8 / 8) |
