# Supplementary material for: No beneficial effect of aerobic whole-body electromyostimulation on lower limbs strength and power – a randomized controlled trial
Source: BMC Sports Sci Med Rehabil. 2024 Jul 2;16:144. doi: 10.1186/s13102-024-00931-4 (PMC11218063; doi:10.1186/s13102-024-00931-4)
Supplement: Supplementary file 2 — Supplementary Material 2. [file 13102_2024_931_MOESM2_ESM.docx]

**Supplement Table 2.** Physical activity and nutritional behavior based on protocols from pre- to post-intervention (mean ± SE).

|  | EG | | | CG | | |
| --- | --- | --- | --- | --- | --- | --- |
|  | **pre** | **post** | ***p*** | **pre** | **post** | ***p*** |
| Energy turnover/ week | n=22  3621±549 | n=22  3085±354 | 0.23 | n=17  3763±618 | n=17  3829±888 | 0.92 |
| Caloric intake [kcal] | n=13  5532±423 | n=13  5453±378 | 0.80 | n=7  6125±612 | n=7  6413±400 | 0.58 |
| Carbs | n=13  584±46 | n=13  598±43 | 0.72 | n=7  652±69 | n=7  683±41 | 0.56 |
| Fats | n=13  235±22 | n=13  237±23 | 0.94 | n=7  272±37 | n=7  292±24 | 0.61 |
| Proteins | n=13  235±79 | n=13  205±18 | 0.08 | n=7  253±19 | n=7  253±13 | 0.99 |
